# Medical Student Note Quality on a Pediatrics Core Clerkship Differs by Service

**DOI:** 10.7759/cureus.44740

**Published:** 2023-09-05

**Authors:** Barbara D Friedes, Ashlyn E McRae, Jareatha Abdul-Raheem, Eric Balighian, William Golden, Amit K Pahwa

**Affiliations:** 1 Pediatrics, Children's Hospital of Philadelphia, Philadelphia, USA; 2 Pediatrics, Northwestern University Feinberg School of Medicine, Chicago, USA; 3 Pediatrics, Johns Hopkins University School of Medicine, Baltimore, USA; 4 Pediatrics, Johns Hopkins University, Baltimore, USA; 5 Internal Medicine - Pediatrics, Johns Hopkins University, Baltimore, USA

**Keywords:** clinical documentation, pediatrics, medical education, subspecialty, communication skills, clinical, electronic medical record

## Abstract

Introduction

Medical students rotate on various clinical disciplines with the same professional goal of learning medical documentation. This study investigated differences between medical student notes on inpatient general and subspecialty pediatric services by comparing note quality, length, and file time.

Methods

In a single-site, observational cohort study, medical students in the Core Clerkship in Pediatrics (CCP) from July 2020 to June 2021 participated in a note-writing didactic course. We compared notes from medical students completing their inpatient assignment on a general pediatric service to those who completed it on a pediatric subspecialty service. Primary outcomes were note quality measured by Physician Documentation Quality Instrument-9 (PDQI9), note length (measured by line count), and file time (measured by hours to completion since 6 AM on the morning of note initiation).

Results

We evaluated 84 notes from 84 medical students on the general pediatric services and 50 notes from 49 medical students on the pediatric subspecialty services. Note quality measured by PDQI9 was significantly higher for general pediatric service notes compared to pediatric subspecialty service notes (p = 0.03). General pediatric service notes were significantly shorter (p < 0.001). We found no difference in file time (p = 0.23).

Conclusion

Medical student notes on pediatric subspecialty services scored significantly lower in quality and were longer compared to general pediatric services, demonstrating the need for a more tailored note-writing curriculum and note template based on service.

## Introduction

Medical students explore various disciplines through clinical rotations in general and subspecialty medicine. This exposure may influence later decisions to pursue subspecialty training [1]. Moreover, subspecialty service exposure during core clerkship rotations potentially improves students’ clinical evaluations without negatively impacting their National Board of Medical Examiners (NBME) shelf performance or final clerkship grade [2].

During clinical clerkships, medical students learn to document their work in the electronic medical record (EMR). This skill is an Entrustable Professional Activity (EPA) that the American Association of Medical Colleges (AAMC) recommends achieving before medical school graduation [3]. Despite this recommendation, one study showed over 35% of third-year medical students’ notes were incomplete, inappropriate, or inaccurate [4]. Additionally, redundancy, such as copy-paste formatting without revisions that reflect changes in clinical reasoning, pervades medical student notes and has been associated with lower United States Medical Licensing Examination (USMLE) step II clinical knowledge scores [5]. These deficiencies may be explained by the lack of formalized education on note writing [6] or the lack of feedback for trainees from clinical faculty [7]. Despite a policy change in February 2018 by the Center for Medicare and Medicaid Services approving the use of medical student notes for billing purposes [8], most medical student notes are still commonly used solely for educational purposes, if at all [9].

While benefits exist in rotating on subspecialty services, medical students may be mentally unprepared for accurately documenting their clinical care on subspecialty rotations due to their paucity of general medicine knowledge to serve as a foundation to gain subspecialty knowledge. Many institutions utilizing medical student notes in patient health records only use those from general ambulatory or inpatient services [9]. Currently, a gap in the literature exists regarding the assessment of medical student note-writing capabilities or needs for subspecialty services. 

At the Johns Hopkins University School of Medicine (JHUSOM), the Core Clerkship in Pediatrics (CCP) developed a note-writing curriculum and note templates to help meet this critical core professional EPA and utilize these notes for billing [10]. The curriculum and note templates were based on previously published data that showed this additional training could improve note quality, decrease note length, and decrease time to note completion for internal medicine residents [11]. Additionally, the CCP, in conjunction with the pediatrics residency program, created a standard workflow for medical student note review, editing, and attesting to be used by general pediatric hospitalist attendings for billing purposes. The implementation of a note-writing curriculum and note template on CCP increased the quality and decreased the length of medical student notes on inpatient general pediatric services compared to the medical student notes written before this intervention [10].

In July 2020, the CCP assigned medical students to inpatient subspecialty services due to a reduction in clinical sites resulting from the coronavirus disease 2019 (COVID-19) pandemic. All students participated in the same note-writing curriculum and received the same note templates. However, a comparison of student documentation on inpatient pediatric subspecialty services (versus inpatient general pediatric services) has not been evaluated. Therefore, we sought to investigate differences between medical student note writing in our inpatient pediatric services by comparing note quality, length, and file time. We hypothesized that the note-writing curriculum would be effective for medical students on general pediatric services, but medical students would have more difficulty clinically documenting in pediatric subspecialty services, resulting in lower-quality notes.

## Materials and methods

This single-site observational cohort study included all medical students enrolled in a six-week required CCP from July 2020 through June 2021. As part of the CCP, all medical students participated in a one-hour didactic session on writing notes in the EMR and were provided a note template, as previously described by McRae et al. [10]. The curriculum for the didactic session and note template was based on previously published data from internal medicine residents [11]. The note template was created utilizing input from fourth-year medical students, CCP leadership, pediatric residents, and faculty from the Division of Pediatrics and Pediatric Hospital Medicine. Medical students were encouraged to use the template while on the pediatrics service. The Johns Hopkins Medicine Institutional Review Board deemed this study exempt (IRB00208342).

This study compared notes from medical students completing their inpatient assignment on a general pediatric service to students who completed their inpatient assignment on a pediatric subspecialty service (cardiology, gastroenterology, hematology, pulmonology, or nephrology). To collect all inpatient notes written by medical students throughout the study period, we created a report in the EMR (Epic ®, Verona, WI) from July 2020 through June 2021. Inclusion criteria consisted of having at least two consecutive inpatient notes authored by the same medical student for the same patient. The first two qualifying notes under these criteria were selected. The first note was used as a reference, with the second note as the single document utilized for grading and analysis. We utilized the first note as a reference in time, as one of the PDQI9 items for scoring is "up to date.” All notes were copied into Microsoft Word (Microsoft Corporation, Redmond, WA) and de-identified of medical student author and patient identifiers.

Using the Physician Documentation Quality Instrument-9 (PDQI9), de-identified notes were graded by two pediatric faculty members (one pediatric hospitalist and one pediatric subspecialist) who regularly instruct medical students on the CCP but were not directly involved with students included in this study [12]. These faculty graded an equal proportion of both general pediatric and pediatric subspecialty notes. The summation of scores from each category of the PDQI9 was used as the total score.

In addition to the primary outcome of quality measured by the PDQI9, we measured note length and file time. We measured note length by manually counting the number of text lines of the note in Epic® using the same computer monitor with the software in the maximum screen mode to mitigate variance in line counting. We determined file time by assessing hours to completion of the note since 6 AM on the morning of note initiation (the beginning of the service for the daytime inpatient teams). This time assessment represented when the medical student last shared their note, allowing the senior residents and attendings to edit and attest the note.

The Mann-Whitney U test was used to compare results between the general pediatric and pediatric subspecialty notes for statistical analysis, with p ≤ 0.05 considered significant. Fisher’s exact t-test was used to compare categorical data. Statistics were completed using Prism GraphPad^©^ (GraphPad Software, Boston, MA).

## Results

**Figure 1:**
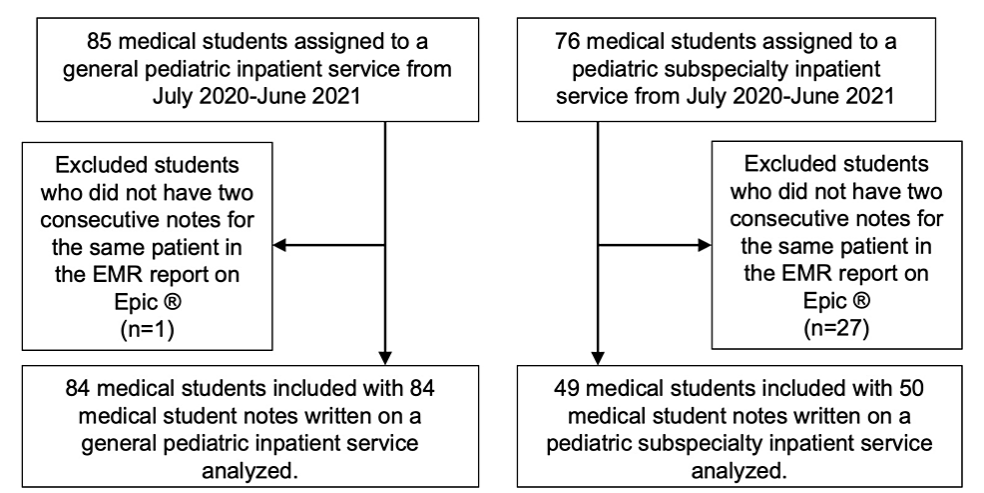
Inclusion and exclusion criteria for medical student notes Medical students who were assigned to an inpatient general or subspecialty pediatric service between July 2020 and June 2021 were eligible for the electronic medical record (EMR) report search in Epic®. Medical students were excluded if they did not have two consecutive notes written for the same patient on one service.

From July 2020 through June 2021, 85 medical students were assigned to the general pediatrics inpatient services and 76 medical students were assigned to the pediatric subspecialty services for the CCP. Based on inclusion criteria, we used 84 notes from the inpatient general pediatric services and 50 notes from the inpatient pediatric subspecialty services (Figure 1). Of the medical students with notes included in the study, 22% (n=24) rotated on both inpatient general pediatric and pediatric subspecialty services, 55% (n=60) rotated on general pediatric inpatient services only, and 23% (n=25) rotated on pediatric subspecialty inpatient services only.

**Figure 2:**
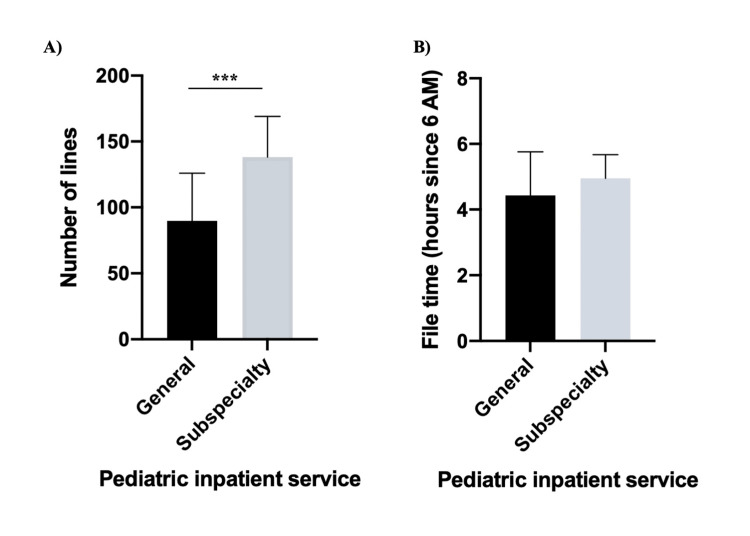
Measurements of medical student note writing on pediatric inpatient services Graphs represent median and interquartile range (IQR). A) General pediatric notes are significantly shorter than pediatric subspecialty notes (p < 0.001). B) There was no difference between file time for the two note types (p = 0.23).

For the primary outcomes, the median note quality score (measured on PDQI9) was significantly higher for the notes on the general pediatric services (total score 35, IQR 33-41) compared to pediatric subspecialty services (total score 34, IQR 31.25-37.75) (p = 0.03) (Table 1). Furthermore, there were two significant differences in the PDQI9 subcategories. Medical students’ notes on the general pediatric service (median 4, IQR 3-4) were significantly more useful than the pediatric subspecialty notes (median 3, IQR 3-4) (p = 0.04). Additionally, medical students’ notes were significantly more comprehensible on the general pediatric service (median 4, IQR 4-4) compared to the pediatric subspecialty services (median 4, IQR 3-4) (p = 0.04). There were no significant differences in the other seven subcategories of the PDQI9. Moreover, general pediatric service notes were significantly shorter (Figure 2) than notes written on pediatric subspecialty services (89.5 lines, IQR 62.5-125.8 vs. 138 lines, IQR 106.3-169; p < 0.001). There was no difference in file time (4.4 hours, IQR 3.3-5.8 vs. 5.0 hours since 6 AM, IQR 3.9-5.7; p = 0.23).

**Table 1:** Physician Documentation Quality Instrument-9 (PDQI9) assessment of medical student notes on general pediatric and pediatric subspecialty inpatient services

PDQI9 component	General pediatric notes (n=84) Median (IQR)	Pediatric subspecialty notes (n=50) Median (IQR)	Mann Whitney U Test P-Value
Up to Date	4 (4-5)	4 (4-4.25)	0.39
Accurate	4 (4-5)	4 (4-5)	0.88
Thorough	4 (3.25-4)	4 (3-4)	0.37
Useful	4 (3-4)	3 (3-4)	0.04*
Organized	4 (4-5)	4 (3-4.25)	0.10
Comprehensible	4 (4-4)	4 (3-4)	0.04*
Succinct	4 (3-5)	4 (3-4.25)	0.08
Synthesized	4 (3-4)	4 (3-4)	0.25
Internally Consistent	4 (4-4)	4 (4-4)	0.64
Total Score	35 (33-41)	34 (31.25-37.75)	0.03*
PDQI9: Physician Documentation Quality Instrument-9, IQR: interquartile range between the 25^th^ and 75^th^ percentile, *p-value < 0.05			

In a subset evaluation, we evaluated how many students utilized the CCP note template provided versus the resident template (note template used by residents). In the general pediatrics service, 70% (n = 59) of medical students utilized the CCP note template in the evaluated note. In the pediatric subspecialty services, significantly fewer medical students (n = 13, 26%) used the CCP note template (p < 0.001). To evaluate if this discrepancy in note template affected our outcomes, we performed the same comparisons of our primary outcomes between the general pediatric and pediatric subspecialty services (Table 2). For the notes written with the CCP template, there were no significant differences between PDQI9 components or the total score between general pediatrics and pediatric subspecialty services. In contrast, for the notes written with the resident template, there were several components of the PDQI9 that scored significantly higher on the general pediatric service, including up to date (p = 0.02), useful (p = 0.007), organized (p = 0.002), comprehensible (p = 0.04), succinct (p < 0.001), and total score (p = 0.001). There were also no differences between file time or note length for either comparison with the CCP or resident template.

**Table 2:** Comparison of medical student notes on general pediatric and pediatric subspecialty inpatient services by the type of note template utilized to evaluate note quality (by Physician Documentation Quality Instrument-9 (PDQI9) assessment), note file time, and note length

	Core Clerkship in pediatrics template notes			Resident template notes		
	General pediatrics (n = 59) Median (IQR)	Pediatric subspecialty (n = 13) Median (IQR)	Mann Whitney U Test P-Value	General pediatrics (n=25) Median (IQR)	Pediatric subspecialty (n=37) Median (IQR)	Mann Whitney U Test P-Value
PDQI9 Up to Date	4 (4-5)	4 (4-4)	0.555	5 (4-5)	4 (4-5)	0.02*
PDQI9 Accurate	4 (4-5)	4 (4-5)	0.913	5 (4-5)	4 (4-5)	0.13
PDQI9 Thorough	4 (3-4)	4 (3-4)	0.225	4 (4-5)	4 (4-4)	0.13
PDQI9 Useful	4 (3-4)	3 (3-4)	0.278	4 (3-5)	3 (3-4)	0.007*
PDQI9 Organized	4 (4-5)	4 (3.5-5)	0.894	5 (4-5)	4 (3-4)	0.002*
PDQI9 Comprehensible	4 (4-4)	4 (3-4)	0.708	4 (4-4)	4 (3-4)	0.04*
PDQI9 Succinct	4 (3-5)	4 (3-5)	0.718	5 (4-5)	4 (3-4)	<0.001*
PDQI9 Synthesized	4 (3-4)	4 (3-4)	0.958	4 (4-4)	4 (3-4)	0.08
PDQI9 Internally Consistent	4 (4-4)	4 (4-4)	0.513	4 (4-5)	4 (4-4)	0.10
PDQI9 Total Score	35 (32-37)	34 (32-38.5)	0.769	39 (35-43)	34 (32-37.5)	0.001*
File time (hours since 6:00 AM)	4.38 (2.78-5.63)	4.78 (3.44-5.65)	0.731	4.73 (3.62-5.93)	4.97 (4.01-5.67)	0.54
Note line count	72 (57-94)	77 (58-131)	0.431	143 (121-162)	150 (118.5-176.5)	0.42
PDQI9: Physician Documentation Quality Instrument-9, IQR: interquartile range between the 25^th^ and 75^th^ percentile, *p-value < 0.05						

## Discussion

To our knowledge, this study is the first to compare notes on general and subspecialty pediatric inpatient services written by contemporaneous medical students receiving the same note-writing curriculum and note templates. We found that overall and in two subcategories on the PDQI9 (useful and comprehensive), the general pediatric service notes were of higher quality than the pediatric subspecialty service notes. Additionally, the notes from general pediatric services were significantly shorter than pediatric subspecialty notes. These findings demonstrate a need to adapt our current note-writing curriculum to address the differences in documentation on pediatric subspecialty services.

The differences in overall note quality and the two categorical scores of “useful” and “comprehensive” may stem from the original goal of the note-writing curriculum. McCrae et al. created the curriculum and note templates for medical students on general pediatric inpatient services and showed this curriculum to be effective for their intended population [10]. Additionally, the curriculum was adapted from a study based on resident notes written on general internal medicine wards, without subspecialty considerations [11]. Furthermore, the note template was created without input from subspecialty faculty or leadership. Therefore, it is not surprising that the note curriculum and note template did not perform as well on various pediatric subspecialty services. Nonetheless, our comparison was confounded because some medical students used a different resident note template on both the general pediatric and pediatric subspecialty services. The comparison of the notes using exclusively the CCP note template did not show any significant differences in note quality; although this may be due to inadequate numbers for analysis (with only 13 CCP templated notes on the pediatric subspecialty service). Interestingly, when comparing all the notes that utilized the resident template, those written on the general pediatric service had significantly better note quality. This evidence supports the success of the didactic curriculum for students on general pediatrics services, which again is expected due to the curricular origins of general pediatric medical education [10]. For medical students to succeed in pediatric subspecialty services, we surmise the note-writing curriculum must be adapted with partnership from subspecialty faculty and leadership.

 Additionally, notes on the general pediatric inpatient services were significantly shorter than those on the pediatric subspecialty service despite no difference in the PDQI9 in the category for “succinct.” This result suggests the pediatric subspecialty notes were not superfluous and, perhaps, needed to include more information for complexities in patient history, diagnostic testing, assessments, and plans. Alternatively, significantly more students on the pediatric subspecialty service utilized the aforementioned resident template, which auto-populates more background information (such as problem list, and diagnostic values), than the CCP standard note template and may skew the pediatric subspecialty group towards a longer note length. Interestingly, when compared by template type (CCP template or resident template), there were no differences in note length between the general pediatric and pediatric subspecialty notes, suggesting note length is not affected by the type of service, but primarily by the note template. Interestingly, despite the longer length of pediatric subspecialty notes, we noted no significant difference in file time, which may be due to note auto-population. The inclusion of these additional auto-populated elements should be discussed in future iterations of the curriculum to create pediatric subspecialty-specific templates to aid medical student learning.

The limitations of this study included a small sample size, a single study site, and a dynamic environment throughout the study period due to COVID-19 that may have affected medical student learning and note writing. Additionally, a small percentage of students (n=24, 22%) in this study completed time on both general pediatric and pediatric subspecialty inpatient services and had both note types included in this study. However, due to the small sample size of this group, this factor is unlikely to affect our analysis. Lastly, the medical students' use of different note templates limits our comparison. All medical students on the Core Clerkship in Pediatrics were instructed to use the note template provided during the note-writing didactic. As students on both the general pediatric and pediatric subspecialty services utilized the resident templates, we hypothesize residents, fellows, or attendings may have suggested using the resident note templates to be more aligned with their resident supervisors.

## Conclusions

Overall, the current note curriculum and standard progress note template at our institution provide adequate training to allow medical students to generate high-quality notes on general pediatric inpatient services. However, they were not as effective in our pediatric subspecialty inpatient services. In the future, the current note curriculum should be adapted to include additional training for subspecialty-specific needs and subspecialty-specific note templates to improve note quality, reduce note length, and potentially increase note writing efficiency for medical students on pediatric subspecialty inpatient services. Future studies should expand the sample size and implement this note-writing curriculum at multiple sites to evaluate the generalizability of these findings for medical students at different institutions.
